# EXPLORING ASSOCIATIONS BETWEEN FRAILTY AND ORAL HEALTH IN COMMUNITY-DWELLING OLDER PEOPLE

**DOI:** 10.1093/geroni/igz038.179

**Published:** 2019-11-08

**Authors:** Babette Everaars, Katarina Jerković - Ćosić, Nienke Bleijenberg, Niek J de Wit, Geert j van der Heijden

**Affiliations:** 1 University of Applied Sciences Utrecht, Utrecht, Utrecht, Netherlands; 2 Julius Center for Health Sciences and Primary Care, University Medical Center Utrecht, Utrecht, Utrecht, Netherlands; 3 ACTA academisch Centrum Tandheelkunde Amsterdam, Amsterdam, Noord-Holland, Netherlands

## Abstract

This study explores associations between frailty and oral health in cross-sectional data of 1,202 community-dwelling older people. Two dichotomous outcomes were used: 1. Potential frailty, using routine primary care data; 2. Self-reported frailty, using a questionnaire. Oral health concerned dental record data and self-reported oral problems. Following exploration of univariate associations, age and sex adjusted multivariate logistic regressions were performed. For potential frailty and self-reported frailty associations were found with dental emergency visit (odds ratio (OR)= 2.0, 95% confidence Interval (CI)=1.33;3.02 respectively OR=1.58, 95% CI=1.00;2.49), experiencing oral problems (resp. OR=2.07, 95% CI=1.52;2.81 and OR=2.87, 95% CI= 2.07), making dietary adjustments (resp. OR=2.66, 95% CI= 1.31;5.41 and OR=5.49, 95% CI= 3.01;10.01). Additional associations were found for self-reported frailty with wearing dental prosthesis (OR=3.33, 95% CI=1.49;7.44) and missing periodontal information (OR=1.56, 95% CI=1.05;2.32). The cross-sectional data of this study show that in community dwelling older people oral health is associated with frailty.

